# Role of amino acid infusion in delayed recovery from neuromuscular blockers

**DOI:** 10.4103/0019-5049.79887

**Published:** 2011

**Authors:** Anju Gupta, Nishkarsh Gupta

**Affiliations:** Pandit Madan Mohan Malviya Hospital, New Delhi, India; 1 Department of Anaesthesiology and Pain Management, PGIMER and Associated Dr RML Hospital, New Delhi, India

Sir,

I appreciate the successful management of a patient with recurarisation[1] due to suspected hypothermia. But we have some concerns regarding the author’s findings and the related interpretation.

Firstly, hypothermia under anaesthesia classically occurs in three phases; namely, early redistribution phase, intermediate linear phase and a plateau phase.[2,3] On induction of anaesthesia, there is a transfer of heat from core to periphery accounting for 0.5–1°C fall in core temperature during the first hour.[2,3] Since surgery lasted 60 minutes (in the reported case only); the reported fall of temperature of 2°C (to 35°C) is a bit too much for a surgery that lasted only 60 minutes. Moreover, ambient temperature[4] of the operating room is the single most important factor determining degree of redistribution hypothermia but there is no mention of it anywhere in the article.[4]

Secondly, patient was given vecuronium bromide at a dose of 0.1 mg/kg (4.5 mg initial dose) and the total dosage was 4.8 mg. considering this, the rationale and timing of 0.3 mg repeat dose of vecuronium is not clear. Also, there is no mention of time elapsed after the last dose of vecuronium bromide and before attempting reversal. Moreover, since a 3°C fall in core temperature doubles the duration of vecuronium bromide,[5] if hypothermia of a degree mentioned was present, than this would have increased the duration of vecuronium bromide. This becomes important because in the presence of hypothermia reversal of neuromuscular block may be difficult.

Thirdly, once patient was found to have inadequate neuromuscular reversal, no effort was made to assess neuromuscular junction function via clinical tests and neuromuscular monitoring. Instead, temperature monitoring was thought of in the first place (that too core temperature via nasopharyngeal probe in an awake patient!).

Fourthly, although amino acids are known to increase internal heat generation, their effect is more evident inter-operatively because anaesthesia induced inhibition of central thermosensors.[6] Their effect is not expected to be the same postoperatively. It is unlikely that hypothermia was corrected by 150 ml of amino acids. Moreover, the basis of giving amino acids at the rate of 100 ml/hour is also not clarified.

So, findings of this case report should be interpreted with caution and we should be careful in using amino acids blindly during the postoperative period for correction of hypothermia.
